# Proposing an Emergency Medicine Ethical Guideline; a Qualitative Study

**DOI:** 10.22037/aaem.v10i1.1391

**Published:** 2022-01-01

**Authors:** Leili Asadabadi, Kamran Soltani Nejad, Atefeh Zolfagharnasab, Mina Mobasher

**Affiliations:** 1 Clinical Research Unit, Shahid bahonar Academic Center, Kerman University of Medical Sciences, Kerman, Iran.; 2 Afzalipour Faculty of Medicine, Kerman University of Medical Sciences, Kerman, Iran.; 3 Department of Library and Medical Information, Faculty of Management and Medical Information Sciences, Kerman University of Medical Sciences, Kerman, Iran.; 4 Department of Medical Ethics and History of Medicine, Faculty of Iranian Traditional Medicine, Kerman University of Medical Sciences, Kerman, Iran.

**Keywords:** Codes of ethics, emergency medicine, practice guideline, physician-patient relations

## Abstract

**Introduction:**

Emergency medicine physicians face major ethical challenges in their practices. Furthermore, they need to be aware of the principles of ethical analysis and clinical decision-making in order to provide quality care. This study aimed to propose professional ethics codes in the emergency medicine department.

**Method:**

This is a qualitative study, which was performed using narrative review and expert panel, and was conducted in three steps, including: literature review and preparation of the initial draft of the ethical concepts, obtaining expert opinions on this initial draft and its validation, and finalizing main ethical components in emergency medicine. In this study, we received the opinions of an expert panel including 10 medical ethicists and 12 emergency medicine specialists using a survey form.

**Results:**

The ethical guide to emergency medicine can be formulated in 34 key ethical concepts, 6 sub-components, and 5 main components including emergency physician-patient relationship, and emergency physicians’ relationships with other professionals, students, researchers, and community.

**Conclusion:**

Emergency care providers need to be familiar with ethical guidelines in order to improve quality of care in emergency departments. The findings of this study suggest that a guideline on patient-physician relationship as well as the emergency physicians’ ethical obligations for other professionals, students, researchers, and community should be developed in line with ethical norms.

## 1. Introduction

Emergency medicine physicians face major ethical challenges in their practices due to criticality of situations in the emergency department as well as the advances of knowledge and technology in health care services. Consequently, they need individual knowledge and group discussions to make decisions. There is a consensus that moral sensitivity is one of the requirements of physicians' practice in emergency medicine settings (1). The ethical responsibilities of emergency physicians in patient care require that they be aware of their professional values, and the principles of ethical analysis. Furthermore, ethical codes are a systematic guide for shaping the ethical behaviors of people (2). Emergency physicians should be aware that there may be controversial ethical challenges in each of the medical procedures (3). The principles, rules, and ethical standards have been developed at the organizational, national, and even global levels and introduced as professional ethical guidelines for many professions, and professionals are obliged to abide by it (4). According to the American Emergency Medicine Association (AEMA) recommendations, emergency medicine practitioners must be actively engaged in professional ethics in order to ensure the provision of optimal care in the process of clinical decision-making. The performance quality of emergency medicine physicians can improve and contribute to the integration of morality-based practices in different situations provided that there are professional ethics standards and they are practiced (5). Moreover, if medical students and residents work according to ethical principles taught to them during their training in emergency medicine, these principles improve their professional behaviors and moral decision-making, and this is critically important in their professional practice in the future. Emergency physicians should engage in educational programs related to ethical reasoning in order to be able to resolve ethical challenges in complicated situations of emergency departments (6). Teaching the emergency staff about ethical dilemmas in emergency medicine and developing or providing access to appropriate resources such as ethical guidelines can be very helpful in managing difficult emergency situations and some aggressive patients and their families, as well as complex emergency situations (7). Furthermore, a moral guide can also be a reliable tool to evaluate the performance of physicians as well as leading to unity of practice and strengthening the sense of responsibility and professional commitment in physicians. This study aimed to review resources, narratively, and clarify key ethical concepts and main components of the ethical issues in the emergency medicine department. 

## 2. Method

***2.1 Study design and setting***

The present study is a qualitative study that was conducted in three main steps. The first step consisted of drafting the early version of the codes of emergency medicine that included three parts: 1) review of texts using content analysis and extraction of ethical concepts, 2) translating ethical concepts into codes and 3) completion of the code list by adding ethical codes of the other countries.

***2.2 Data gathering***

**Figure1:**
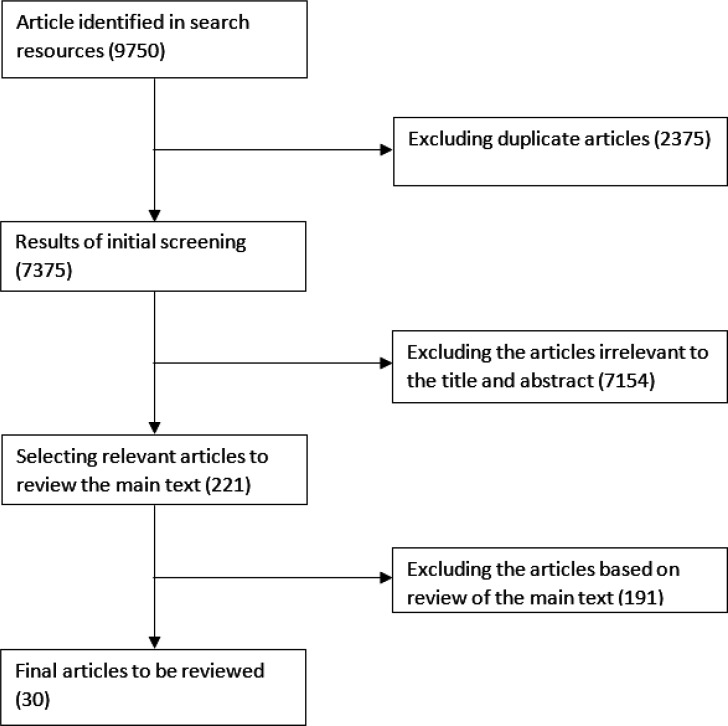
The flowchart of the extracted articles

In order to extract ethical concepts, a search in the three international databases Web of Science, PubMed and Scopus was conducted using the keywords ethics, code of ethics, emergency and EMS in the title and abstract fields without any time limit until the end of 2020. Also, a search was conducted in the Persian language databases including magiran and SID with the keywords including emergency medicine, ethics, medical etiquette, education. In addition, the phrase code of ethics for emergency was used in the Google search engine and the websites of the Ministries of Health in different countries in order to access relevant documents to provide codes of ethics related to emergency medicine in these countries. These documents are important sources of information that can make important contributions to a review, may reduce publication bias, and increase reviews' comprehensiveness. Figure 1 shows the process of extracting relevant articles (Figure 1).

Two members of the research team checked on the credibility of the data source and its usability for our study using common sense and valid search engines. We studied the articles to identify the ethical concepts that are mentioned in a form of behavior (so as to indicate the engamenet in or abandonment of a specific behavior) related to the emergency department. The ethical concepts were merged and categorized. After achieving data saturation and the literature review was stopped. Following this step, ethical concepts became a code of ethics. Otherwise, it would have been written in form of do’s and dont’s that emergency medicine specialists should oblige. Furthermore, to ensure the comprehensiveness of the obtained codes, the ethical codes related to the emergency medicine profession were collected from four countries based on availability and ease of access. Finally, the first draft of the code of ethics was ready to enter the next step.

In the second step of this qualitative study, we used expert sampling, a purposive sampling technique, to include only those with expertise in a certain area (emergency medicine and medical ethics). In fact, these experts are purposefully as different from each other as possible to help in identification and selection of information related to the ethical issues in emergency medicine practices. We try to reduce the biases in this study as much as possible by using this sampling. Our experts were emergency medicine specialists and medical ethicists with different backgrounds from different universities of medical sciences in Iran (Kerman, Qom, Tehran, Rafsanjan, Mashhad). They announced that they did not have any conflict of interest.

In this step of the study, we conducted a survey of experts by emailing them a survey form. The compiled text was sent to ten medical ethicists. According to five of their comments, the necessary modifications were carried out in the initial text by the medical ethicist in our research team. The second text was reread with members of the research group and the required modifications were made. The third version of the compiled text was sent to twelve emergency medicine experts through email and 6 of them sent their comments back. Verbal and written explanation was given to these experts. Also, in the survey step, in order to complete the validation of the obtained codes, experts on the subject were asked to express their views on the compiled text in terms of the clarity of the content of each code and its feasibility. The agreement of medical ethicists in the first stage and the agreement of emergency medicine specialties in the second stage produced data saturation. Therefore, the depth and volume of data was suitable. 

In each step, the completed forms were reviewed, and based on the received suggestions, the necessary changes were made in the forms and writing styles of some codes. Therefore, some codes were combined, adjusted, or deleted, and the opinions of the above-mentioned expert groups were summarized. 

***2.3 Statistical analysis***

Finally, we could clarify the main components by placing similar ethical concepts into one subcomponent by applying the technique introduced in Sandolowski study (8). In this way, the final set of proposed ethical codes in emergency medicine was formulated as the final edition and prepared to be presented to relevant authorities. In this study, we introduced key ethical concepts and main components of the ethical guidelines developed in emergency medicine.

***2.4 Ethical consideration***

This study was approved by Kerman University of Medical Sciences in 2019 with the ethics code (IR.KMU.AH.REC.1398.015) obtained from the National Ethics Committee in Biomedical Research. 

## 3. Results

We extracted 9750 documents to review their titles and abstracts and selected 221 documents for the final review. Of the selected ones, 30 documents were used to compile the codes (Figure 1), most of which were related to the United States (12 documents) and Iran (5 documents), respectively. There were two documents related to the Philippines. Articles (one for each country) were from New Zealand, the United Kingdom, Turkey, Saudi Arabia, Pakistan, and Canada. Also, five documents were co-written (Table 1).

Ethical codes of the countries showed that the general responsibilities of emergency physicians all over the world were considered the responsibility of emergency physicians toward patients’ rights in terms of obtaining informed consent, maintaining privacy and patients’ best interests as well as the social responsibility of emergency physicians and their responsibility toward their colleagues and the community. The ethical concepts are mentioned in a form of behavior (so as to indicate the engamenet in or abandonment of a specific behavior) related to the emergency department

Furthermore, results of the first and second step of our study showed 34 key ethical concepts in the scope of emergency medicine physicians’ practice (Table 2).

According to the results, guidelines that generally apply to ethical compliance in emergency medicine include the principles of respect for autonomy, beneficence, non-maleficence, and justice in medicine. Furthermore, the importance of strengthening ethical virtues in the performance and flexibility of emergency physicians should be emphasized in triage and in the complex challenges and situations of emergency medicine. Therefore, paying attention to the prevailing values in emergency medicine such as adherence to the six professional behaviors including altruism, responsibility, honesty and integrity, respect for others, excellence, and justice was very important. In addition, work conscience, discipline, good mood, attention to the patient and her/his wishes, making trust, adherence to rules and regulations, and acceptance of responsibility for error were important values in emergency medicine. Finally, the ethical concepts were categorized in five main components and 6 sub-components in this area (Table 2). 

## 4. Discussion

In this study, the general structure of the necessary ethical concepts in Iranian emergency medicine were proposed based on the opinions of experts on the subject and the concepts in the articles and the structure of codes in other fields of medical sciences in Iran as well as other countries. According to the results of this study, the general structure of the proposed ethical guideline of emergency medicine included five main components, the relationship between the emergency physician and the patient, other professionals, students, research group, and the community.

One of the important concepts of the present study for emergency physicians was considering the interests of emergency patients, which is in the results of other studies and codes from other countries (9-16) as well as the guide provided by the American Emergency Medicine Association (5). The emergency physicians need to pay more professional attention to their patients’ interests in emergency departments. This principle can indicate paying attention to the patient's pain, and physical and mental sufferings that should be reduced by physicians as much as possible.

Another suggestion of the present study for emergency physicians is to observe non-maleficence to patients, which was also reported in the results of similar studies (6, 13, 15-18) and the other codes (5, 14). There are degrees of risk or side effects in many emergency cases due to the excessive overcrowding and complex conditions prevailing in the emergency department; therefore, emergency physicians are obliged to assess the condition and the possibility of harming patients and make a suitable decision.

Trying to enhance the knowledge and skills of emergency physicians was one of the ethical codes, which can also be identified in the other studies (9, 12, 19, 20). Improving physicians’ knowledge and skills can be classified as one of the codes that significantly affect other concepts in this study, such as benefit for patients, non-maleficence, improving the quality care, and attention to students’ education. When the emergency specialist increases his/her scientific and practical knowledge, he/she can certainly provide better and more effective care for patients. Hence, emergency specialists should pay special attention to this issue.

The results of similar studies (10, 11, 16, 19, 21, 22) and the ethical guideline provided by the American Society of Emergency Medicine (5) emphasized the impartial performance and fair behaviour of emergency physicians. Furthermore, emergency physicians must deal with issues related to the distribution of scarce health care resources and decide which treatments the patients should receive and what facilities they should use in the emergency room due to the special conditions of the emergency department and the hospitalization of patients with unstable vital signs and lowered level of consciousness. So, it is necessary to practice with justice and fairness in the emergency department.

Respect for Patient Autonomy was another code of this study, which is a concept found in many studies (9-14, 16, 18-20, 23) and a guide provided by the American Society of Emergency Medicine, and was also pointed out in the other studies from the United States, New Zealand, and Canada (5, 13, 15). Of course, observing this code for emergency physicians is a little more difficult than physicians in other departments and requires more effort due to the emergency conditions of patients, the risk of patients’ mortality and morbidity, and time constraints in this department. Emergency specialists should try to provide patients with complete information and help their family members decide about the treatment as much as possible.

Improving the quality of patients' care was selected as one of the ethical concepts in the codes of ethics in emergency medicine. This finding has been noted in other researches (9, 12, 19) and other ethical guidelines (5, 13, 15). In fact, improving the quality of patients’ care in this ward will reduce the patients’ mortality and morbidity more than other wards due to the nature of the emergency department.

Another ethical concept in this research was paying attention to students ' training, which is a code of ethics present in the other ethical guidelines of emergency medicine (9, 19, 20). It is necessary that the clinical staff members of emergency departments, in addition to their comprehensive efforts to save patients' lives, not neglect the educational issues related to all groups of students. Because, the training process for interns and students of other related fields such as nursing and emergency medicine assistants is performed in the emergency department of public hospitals. Eventually, these trainees will serve independently in some remote and sometimes deprived areas as the first line of treatment, so, if they receive the more practical and comprehensive training during their studies, they will definitely provide better services, which can certainly lead to reduced medical malpractice and patient mortality and morbidity.

Control of violence in emergency departments was another ethical concept of this research, which is also present in the results of similar researches (11, 12, 18, 20). This issue is important for emergency specialists due to the stressful and complex situations in the emergency departments and the low tolerance of the patients and their companions due to the occurrence of various accidents. The emergency medicine professionals should have more patience against verbal violence of patients and their companions and facilitate the treatment process through appropriate behavior with patients and other colleagues.

Resource allocation consideration and giving enough knowledge about this issue was another research concept that was present in the results of other researches (5, 11, 19, 24). Considering the limited resources in the emergency department, especially in hospitals in developing countries, it is necessary for emergency specialists to have a good relationship with staff and university officials to meet their demands easily. Also, when they are restricted or under adverse conditions, they should request for assistance in order to protect patients’ lives.

Protecting the life of every human being in any position and situation is the duty of the emergency medicine physician. The ethical responsibilities of emergency medicine physicians in their practice and care requires them to be aware of their beliefs, as well as the basis and principles of ethical analysis and decision-making. Ethical rituals (or codes) are systematic guidelines to form moral behaviors. Emergency medical professionals have the responsibility to decide and operate based on professional values in their career.

Since emergency medical practitioners are one of the most important groups of service providers who have a significant impact on the quality of healthcare provision, their compliance with professional ethics will also be an effective factor in improving the quality of care. In addition, a codified ethical guideline can provide a reliable tool for evaluating physicians' performance for regulatory organizations.

**Table 1:** Bibliographic profile of final included studies

Data gathering	Subjects	Study design	Language	Type	Country	Year	First Author
-	-	-	English	Statement	New Zealand	2020	New Zealand Medical Association
Literature review	-	Qualitative	English	Review article	Saudi Arabia	2019	Qusai Talat Alwaznah
Questionnaire	Staff nurses and physicians	Quantitative	English	Article	Canada	2018	Keith A Colaco
-	-	-	English	Book	-	2018	Helen Askitopoulou
-	-	-	English	Practice Guideline	USA	2017	Diane Gurney
-	-	-	English	Statement	USA	2017	American College of Emergency Physicians
-	-	-	English	Statement	Philippine	2017	Philippine Medical Association
In-depth interview	ED physicians	Qualitative	English	Article	Pakistan	2015	Waleed Zafar
Interview; focus group	Nurses	Qualitative	English	Article	-	2014	Maria F Jime´nez-Herrera
Questionnaire	emergency care providers	Quantitative	English	Article	Turkey	2014	Müesser Özcan
	Ambulance professionals	Quantitative	English	Article	-	2014	Anders Bremer
-	-	-	English	Guideline	USA	2013	Torben K. Becker
-	-	-	English	Book	-	2012	John Jesus
Review of Affordable Care Act 2010	-	Qualitative	English	Review article	USA	2012	Catherine A. Marco
Review	-	Qualitative	English	Review article	-	2009	Gregory Luke Larkin
Literature review	-	Qualitative	English	Review article	UK	2007	Khim Horton
Review	-	Qualitative	English	Review article	USA	2007	Rita Sommers-Flanagan
Review	-	Qualitative	English	Review article	Iran	2007	Bagher Larijani
Review	-	Qualitative	English	Review article	USA	2006	Kenneth V. Iserson
Review	-	Qualitative	English	Review article	Iran	2005	Bagher Larijani
-	-	-	English	Statement	Canada	2018	Canadian Medical Association
Review	-	Qualitative	English	Review article	USA	1999	John Brown
Review	-	Qualitative	English	Review article	USA	1998	James Adams
Questionnaire	Emergency medical technicians	Quantitative	English	Article	USA	1996	Bernard Heilicser
Review	-	Qualitative	English	Article	USA	1991	Kenneth V. Iserson
-	-	-	English	Statement	USA	1991	ACEP Ethics Committee
Review; focus group	-	Qualitative	English	Article	Iran	2013	Akram Izadikhah
Review; interview; focus group	Nursing professionals	Mix method	Persian	Thesis	Iran	2011	Mohsen Shahriari
Review	-	Qualitative	Persian	Article	Iran	2011	Amir Ahmad Shojaee
Review; focus group	Nursing professionals	Qualitative	Persian	Article	Iran	2010	Sodabeh Jolaee

**ED: emergency department.**

**Table 2:** Components of professional ethics in emergency medicine

Main Component	Sub-component	Key ethical concepts
The relationship between the emergency physician and the patient	Patient’s rights	attention to the protection of the patient's interests and privacy according to laws and regulations benefits or risks of treatments patient’s decision-making capacity and consent patient’s transfer to other departments in accordance to laws and regulations paying attention to the patients when they refuse treatment attention to the rights of vulnerable patients and minorities attention to the correct principles of resource allocation
The relationship of emergency physicians with other professionals	-Rights of collegues -Individual rights	paying attention to mutual communication and facilitating these relations in terms of protecting patients' interests not having prejudices choosing counselors correct transfer of patients’ care responsibilities paying attention to patients’ disabilities proper use of care protocols coordination with personnel and maintaining dignity and personality preventing deception and fraud and fighting against these problems access to whose right to be supported in emergency challenges non-acceptance of gifts
Relationships with students, interns, and other learners	-Patient’s rights -Student rights	the emergency physician is a role model for learners closely monitoring the moral performance of learners providing feedback to them paying attention to the interests of patients in educational centers and obtaining their consent
Relations with research groups	-Patient’s rights -Research Ethics	following the ethical guidelines in general and specific research observing the main ethical requirements for research studies taking responsibility for the protection of participants’ rights gaining knowledge of the law and regulations on prevention of scientific fraud active participation and supervision in research
The relationship between the emergency physician and the community	-Patient’s rights -Community rights	being active in relation to legal, regulatory, organizational, and educational measures non-discrimination and providing emergency services for all members of society participating in continuous education activities and trainings necessary for the community being aware of strategies for saving resources proper cooperation with pre-hospital staff making the right triage decisions correct action against domestic violence and abuse training to improve the health and safety of the community

## 5. Limitation

 In the present study, the code of ethics published by the Emergency Medicine Association of some countries such as the United States has been considered, which has been cited in many articles and studies of the other countries. But there was limited access to medical codes of emergency medicine in other countries. Comparative research between public and private hospitals is also proposed to take place in the future so that the results can be used for making policies in this area. Moreover, it is better to assess the views of a larger group of experts to finalize the general structure of the ethical codes of emergency medicine in Iran.

## 6. Conclusion

Moral sensitivity is one of the requirements of professional practice in emergency medicine settings. Thus, a codified ethical guideline can establish an applicable policy for these specialists to increase their practical abilities and provide better, and more effective care for patients. In this regard, the findings of this study sheds light on a more rational solution to the ethical issues in emergency medicine physicians’ relationship with patients, other professionals, students, researchers, and communities.

## 7. Declarations

### 7.1 Aknowledgment

This article is the result of a part of the dissertation by Kamran Soltaninejad in the emergency medicine specialty course in Kerman University of Medical Sciences in 2020, approved by Kerman University of Medical Sciences in 2019 with ethics code (IR.KMU.AH.REC.1398.015) obtained from the National Ethics Committee in Biomedical Research. The authors wish to express their deepest gratitude and warmest appreciation to all the medical ethicists and emergency medicine specialists who in any way have contributed and inspired the researchers.

### 7.2 Financial support

None

### 7.3 Conflict of Interest

The authors stated that there was no conflict of interest.

### 7.4 Author contribution

Study concept and design was performed by Mina Mobasher. Searching the articles and other documents was performed by Atefeh Zolfagharnasab. Considering the documents, extracting the articles were done by Mina Mobasher, Leli sadabadi and Kamran Soltani Nejad. Content analysis, extracting the ethical concepts, and drafting of the ethical codes were done by Mina Mobasher, Leli sadabadi, Kamran Soltani Nejad and Atefeh Zolfagharnasab. All authors reviewed the final draft of this article. Study supervision was performed by Mina Mobasher.
